# *Bordetella pertussis* in hospitalised children and adolescents: the impact of vaccination delay, Tuscany, Italy, 2016 to 2024

**DOI:** 10.2807/1560-7917.ES.2025.30.34.2500062

**Published:** 2025-08-28

**Authors:** Francesco Nieddu, Francesca Quaranta, Marina Vignoli, Matilde Peri, Valentina Guarnieri, Caterina Pelosi, Emanuela Ferraro, Lorenzo Lodi, Silvia Ricci, Giuseppe Indolfi, Chiara Azzari, Maria Moriondo

**Affiliations:** 1 Meyer Children's Hospital, Scientific Institute for Research, Hospitalization and Healthcare (IRCCS), Florence, Italy; 2 Department of Health Sciences, University of Florence, Florence, Italy; 3 Department NEUROFARBA, University of Florence, Florence, Italy

**Keywords:** *Bordetella pertussis*, whooping cough, outbreak, vaccination, vaccination delay

## Abstract

**BACKGROUND:**

Pertussis is a highly contagious respiratory infection caused by *Bordetella pertussis*. Vaccination against pertussis is included in the Italian vaccination programme with three doses administered at 3, 5 and 11 months, booster doses at 6 and 12–18 years, and every 10 years. Vaccination coverage in Tuscany is high among infants (97.7% vs 94.7% national average at 24 months) and adolescents (75.8% vs 68.4% national average at 16 years).

**AIM:**

We aimed to investigate case numbers, vaccination status and time points for vaccination of children and adolescents hospitalised for pertussis.

**METHODS:**

We collected data on children and adolescents aged ≤ 16 years and hospitalised for laboratory-confirmed pertussis in 2016–2024 at a tertiary hospital in Tuscany.

**RESULTS:**

A total of 384 children and adolescents were hospitalised in 2016–2024. Annual case numbers increased from an average of 28.2 cases in 2016–2019 to 259 cases in 2024, with 136 (52.5%) cases in adolescents. Of the 107 cases aged 12–16 years, 93 (86.9%) were unvaccinated or had not received the second booster. A considerable time gap between the earliest eligible day for vaccination and hospitalisation was observed. In infants, a median of 31 days (interquartile range (IQR): 10–131 days) was noticed for the first dose, 44 days (IQR: 22–70 days) for the second and 53 days for the third. In 12–16-year-olds, a median of 395 days (IQR: 236–717) was seen for the second booster.

**CONCLUSION:**

Administering adolescent booster doses earlier, vaccinating at the earliest eligible time points, and promoting timely vaccination through targeted communication campaigns may reduce pertussis-related hospitalisations.

**Key public health message**

**What did you want to address in this study and why?**

Whooping cough or pertussis is a highly contagious respiratory illness caused by *Bordetella pertussis* and preventable by vaccines. We investigated how many children and adolescents aged ≤ 16 years were hospitalised for pertussis in Tuscany, Italy, from 2016 to 2024 and if and when they were vaccinated.

**What have we learnt from this study?**

From 2016 to 2019, every year, an average of 28 children and adolescents were hospitalised for pertussis. In 2024, 259 cases were hospitalised, just over half of them (n = 136) were adolescents. None of the mothers of the 20 hospitalised infants under 2 months old, and too young to be vaccinated, had received the recommended pertussis vaccination during pregnancy, despite it being offered free of charge.

**What are the implications of your findings for public health?**

Childhood vaccination coverage is measured at 2 years of age, but this metric does not capture delays in recommended dose administration. Timely vaccination can protect from severe disease and hospitalisation. A targeted communication campaign aimed at families and healthcare providers should highlight the importance of both getting vaccinated and doing so as early as possible to further reduce cases and hospitalisations among children and adolescents.

## Introduction

*Bordetella pertussis* (Bp) is the causative agent of pertussis (whooping cough), a widespread and highly contagious respiratory infection. Although the bacterium can infect individuals of all ages, infants are particularly vulnerable to severe manifestations of the disease, which can occasionally be fatal [1]. Pertussis exhibits a well-documented seasonal pattern, with case numbers typically increasing during the spring and summer months [2]. Pertussis can be prevented by vaccination, and Tuscany follows the National Immunisation Plan of Italy, recommending pertussis vaccination at 3, 5 and 11 months, with boosters at 6 years, 12–18 years, and every 10 years thereafter alongside diphtheria and tetanus.

Tuscany region has a high coverage rate for pertussis vaccination. In 2023, 97.7% of 2-year-olds were vaccinated, more than the Italian national average of 94.8%, and at 16 years of age, coverage was 75.8% compared with 68.4% nationally [3]. Nonetheless, even in regions where vaccination coverage is high, *B. pertussis* continues to circulate, and outbreaks may still occur [4-6].

The essential role of adherence to the vaccination programme in preventing severe disease and reducing transmission is well recognised. Communication campaigns aiming at motivating healthcare professionals and caregivers, and at improving vaccine acceptance, have been pivotal. Over recent decades, several initiatives have been undertaken to increase vaccination coverage, such as the distribution of information toolkits to families by primary care physicians or paediatricians and targeted vaccination awareness campaigns within specific communities [7-10]. However, while the overall vaccination status according to the immunisation schedule is often monitored, the importance of being vaccinated promptly, on the earliest eligible day, is probably underestimated. Many public health interventions do not specifically emphasise the urgency of early immunisation.

We aimed to investigate, in a region with high pertussis vaccination coverage, adherence to the recommended vaccination schedule and the time interval, if any, between the earliest eligible date for vaccine administration and the date of hospitalisation for pertussis.

## Methods and definitions

### Vaccination programme against pertussis in Tuscany

The vaccination programme in Tuscany includes three doses of hexavalent (diphtheria–tetanus–pertussis (DTP)–polio–*Haemophilus influenzae* type b (Hib)–hepatitis B (HBV)) vaccine at 3, 5 and 11–12 months of age, followed by a booster dose at 6 years and a booster dose at 12–18 years with a quadrivalent vaccine (DTP–polio). Thereafter, a booster is recommended every 10 years, in combination with diphtheria and tetanus vaccinations. All vaccinations included in the programme are provided free of charge.

A child is considered formally compliant for the first dose at the age of 1–2 months, regardless of whether the dose has yet been administered. Compliance for the second dose is defined as being within the eligible time frame for that dose (the fifth month of life), even if the vaccine has not yet been received. Similar criteria were applied for subsequent doses. For instance, an adolescent aged 12–16 years is defined as formally compliant if all previous doses have been administered, even if the adolescent booster, scheduled at 12–18 years, has not yet been received.

### Study design and case definitions

**Figure 1:**
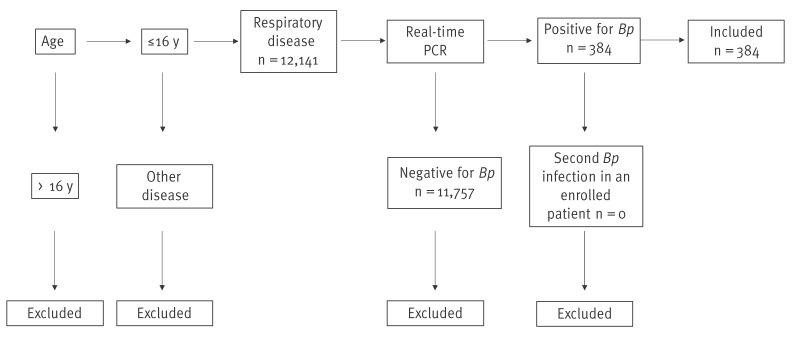
Flowchart of inclusion and exclusion criteria in the study of hospitalised pertussis cases aged ≤ 16 years, Tuscany, Italy, 2016–2024

We conducted a retrospective observational study of all pertussis-related hospitalisations from 2016 to 2024 in a tertiary university hospital in Tuscany (Figure 1).

*Bp*: *Bordetella pertussis*.

We defined a pertussis case as a patient aged ≤ 16 years and admitted to hospital with a clinical diagnosis of pertussis, subsequently confirmed by detection of the bacterium from an anterior nasal or oropharyngeal swab with real-time PCR, in accordance with recommendations of European Centre for Disease Prevention and Control (ECDC), as previously described [11,12]. Before 2021, we used primers, probes and procedure described by ECDC [11]. Since 2021, the Allplex Respiratory Panel 4 (Seegene, Seoul, South Korea) has also been used. All testing was performed in the Laboratory of Immunology, Microbiology and Molecular Surveillance of the Meyer Children’s Hospital IRCCS in Florence, where PCR-based surveillance of all paediatric respiratory infections in Tuscany is routinely conducted. Patients negatively tested for *B. pertussis* were excluded from the study. Each patient was included only once in the study. The Meyer hospital has annually approximately 8,800 inpatient discharges, 33,700 day hospital visits, 40,000 emergency department visits and 867,000 outpatient services.

### Vaccination status

Vaccination status was retrieved from the unified digital vaccination registry of Tuscany region, which is updated daily by family paediatricians and vaccination centres. For each patient, the time interval between the first eligible day for vaccine administration (when the vaccine could have been given but was not) and the onset of disease was calculated. Thus, the delay was defined as the number of days between the first eligible vaccination date and disease onset. At the time of admission, maternal pertussis vaccination status was also recorded for children aged < 3 months.

### Statistical analysis

Descriptive statistical analyses were performed using SPSS version 21 (https://www.ibm.com/products/spss-statistics). Continuous variables were expressed as medians with interquartile ranges (IQR), and categorical variables were expressed as frequencies and percentages.

## Results

### Descriptive analysis of pertussis patients

**Figure 2:**
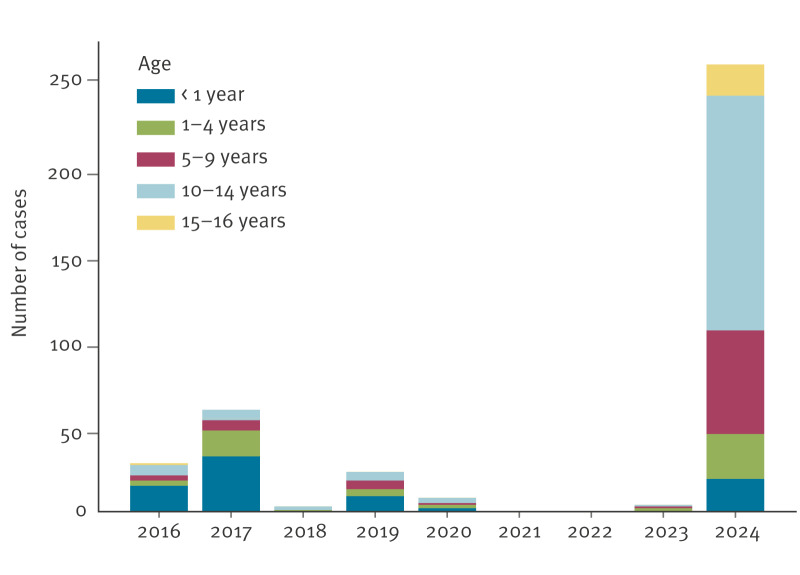
Hospitalised pertussis cases aged ≤ 16 years, by age and year, Tuscany, Italy, 2016–2024 (n = 384)

From January 2016 to December 2024, 384 children and adolescents aged ≤ 16 years with laboratory-confirmed pertussis were hospitalised at Meyer Children’s Hospital. The age distribution is shown in Figure 2.

*Bp*: *Bordetella pertussis*.

From 2016 to 2023, the median age of the cases was 1.6 years (IQR: 0.2–7.1 years; mean: 4.0 years). During this period, 58 (46.4%) of 125 cases were infants (aged ≤ 1 year).

**Figure 3:**
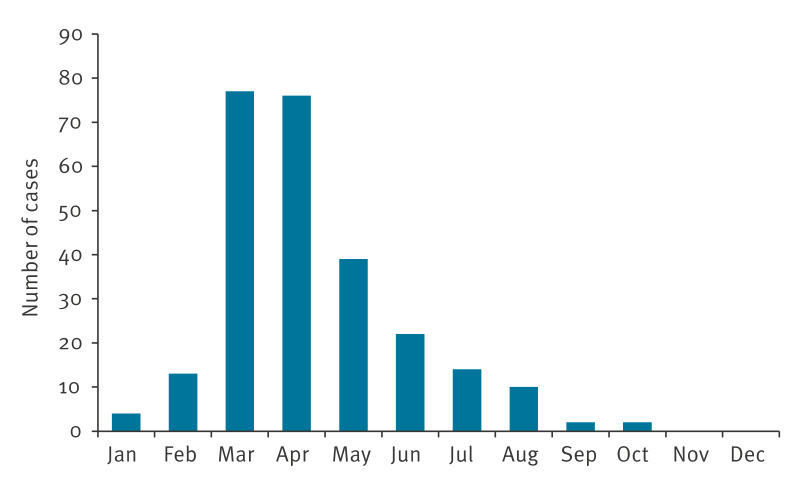
Hospitalised pertussis cases aged ≤ 16 years, by month, Tuscany, Italy, 2024 (n = 259)

In 2024, a rapid increase in pertussis-related hospitalisations was observed, starting in January, peaking in March and April, and declining to no hospitalisations by November (Figure 3).

Compared with 2016–2019, there was a ninefold increase in admissions in 2024, rising from a mean of 28.2 to 259 cases per year. Notably, 51.7% of the 259 cases in 2024 were adolescents (aged 10–16 years). The median age of the cases increased from 1.6 years in 2016–2023 to 11.1 years in 2024 (IQR: 7.8–13.0 years; mean: 9.8 years). In 2024, most (136/259, 52.5%) cases were aged 10–14 years, while only 7.3% (19/259) were infants.

### Vaccination status of pertussis cases

Vaccination data were available for 368 (95.8%) of 384 cases. Among the 77 cases aged < 1 year, 20 (26.0%) were aged < 2 months and therefore too young to be vaccinated. None of their mothers had received the recommended pertussis vaccination during pregnancy, despite it being offered free of charge. Of the 57 infants eligible for vaccination, 29 had not received any dose. The mean delay between the first eligible vaccination date and hospitalisation was 78 days (median: 31 days; IQR: 10–131 days). Eleven infants had received one dose and were eligible for the second but had not yet received it; in this group, the mean delay was 61 days (median: 44 days; IQR: 22–70 days). Three infants had received two doses and were eligible for the third but had not yet received it, with a mean delay of 46 days (median: 53 days). Fourteen infants had received all doses for which they were eligible.

Vaccination status was not documented for 16 (5.2%) of 307 cases aged > 1 year. Among the remaining 291, vaccination was not compliant with the recommended schedule for 67 cases (23.0%): 51 (17.5%) were unvaccinated, 4 (1.4%) had received one dose and 12 (4.1%) had not the full vaccination for their age (third dose or booster).

Of the 178 adolescent cases, 71 (39.9%) were aged 10–12 years and received the booster at 6 years but were below the recommended adolescent booster (fifth dose) age of 12–18 years. Of the 107 cases aged 12–16 years, vaccination status could not be confirmed for 9 (8.4%), 5 (5.1%) were unvaccinated, 88 (89.8%) had received four doses and 5 (5.1%) had received five doses. Among the 88 who had received four doses and were eligible for five doses, the mean of the time gap between the first eligible day for vaccination and disease onset was 542 days (median: 395 days; IQR: 236–717 days).

**Figure 4:**
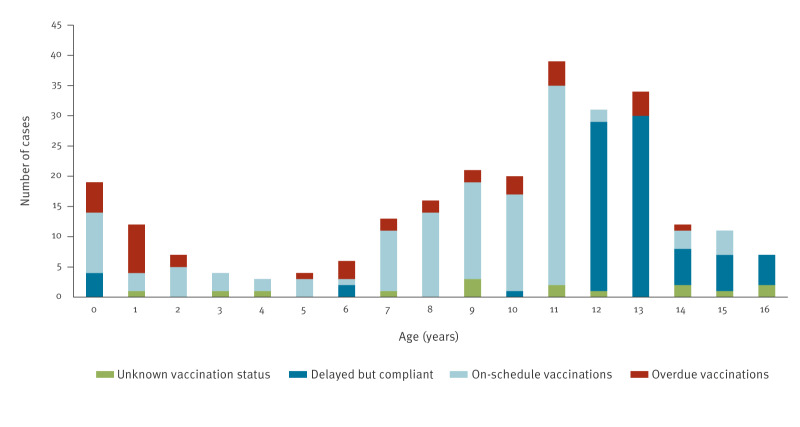
Vaccination status of hospitalised pertussis cases aged ≤ 16 years, by age, Tuscany, Italy, 2024 (n = 259)

Vaccination status during the 2024 outbreak is summarised in Figure 4. Data indicate that although most adolescents appeared to be formally compliant (within the scheduled window of 12–18 years), 95% had not yet received the adolescent booster at the time of the hospitalisation.

In Tuscany, vaccine against pertussis is administered at 3, 5 and 11–12 months of age, followed by booster doses at 6 years and at 12–18 years.

### Age and duration of hospitalisation

**Figure 5:**
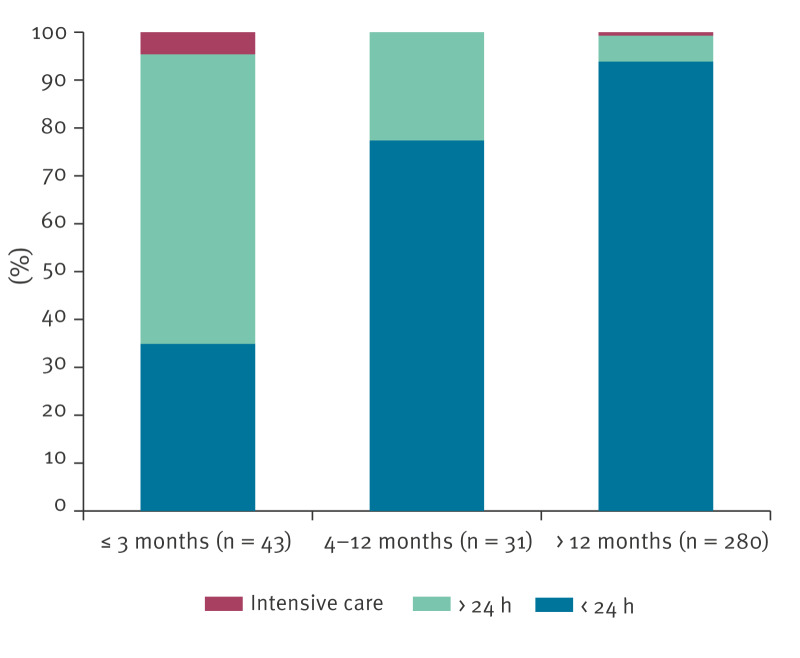
Hospitalisation of pertussis cases aged ≤ 16 years, by age and length of hospital stay, Tuscany, Italy, 2016–2024 (n = 384)

The reason for hospitalisation was persistent cough or lower respiratory airways disease. Of the 384 cases, 150 (39.1%) were hospitalised for < 24 h and discharged after laboratory confirmation of the diagnosis. All cases received antimicrobial treatment, either azithromycin or clarithromycin. The duration of hospitalisation and the proportion of patients requiring high-intensity care is shown in Figure 5.

## Discussion

Following a period from mid-2020, coinciding with the implementation of social distancing measures to contain the COVID-19 pandemic, until the end of 2023 during which no cases were recorded, a marked and rapid increase in pertussis cases was observed at the beginning of 2024, mirroring observations in other countries [4-6].

As also reported in several European countries, such as Denmark, Spain and France, the 2024 outbreak in Tuscany affected adolescents, while the proportion of cases in infants was lower compared with previous outbreaks [13-15].

Most adolescent cases had been appropriately vaccinated in early childhood in accordance with the Italian immunisation schedule, suggesting that waning immunity — rather than vaccine refusal — was the principal determinant of pertussis in this age group. In our study, the cases were seldom vaccinated during the earliest days they were eligible for vaccination. The median interval between the first eligible vaccination date and the onset of pertussis was 31 days for the first dose, 44 days for the second and 53 days for the third. However, our data suggest that in most hospitalised pertussis cases, vaccination had not been administered at the earliest time points. It is likely that timely vaccination could have prevented hospitalisation in many of these cases. Among adolescents, the average time between the first eligible date for the booster (fifth dose) and the onset of disease exceeded 1 year. According to the current Italian vaccination schedule, the adolescent booster can be given at the age of 12–18 years. However, we consider this a missed opportunity for protection as 93 of our 98 cases aged 12–16 years had not received the booster at the time of hospitalisation.

We do not know how common delaying vaccination is and what the reasons are. Shortening this interval and recommending timely administration shortly after the 12th birthday could reduce hospitalisations. A similar interpretation can be drawn from data on younger children. In Tuscany, as in other European countries, childhood vaccination coverage within the first 2 years of life is high. The importance of this study is to examine not only whether vaccinations were administered, but also whether they were administered at the earliest possible time within the recommended schedule.

Contrary to our findings, an eightfold increase in pertussis cases among children aged < 2 years was seen in the 2024 outbreak in southern Italy [16]. Our data align more closely with the observations in other European countries [13-15], with a significant rise in cases among adolescents, particularly those aged 10–16 years during 2024 compared with the pre-pandemic period. Only a modest increase in cases among infants was observed, with 19 cases recorded in 2024 vs an annual average of 14 pre-pandemic. The proportion of cases in children aged < 2 years dropped sharply, from 50% in the pre-pandemic years to 4.9% in 2024.

Our findings also confirm earlier studies that identified age and vaccination status as key factors in determining disease severity [17-20]. Notably, infants required longer hospital stays, which may be considered a proxy indicator of severity.

A limitation of this study is that we could not correlate the type of vaccine received with the risk of hospitalisation. Over the study period, different hexavalent and quadrivalent (DTP–polio) vaccines were used due to frequent changes in procurement tenders. Consequently, it was not possible to determine whether the number of vaccine components (2, 3 or 5) influenced outcomes.

Vaccination coverage in Italy varies by region, and Tuscany has one of the highest coverage rates for pertussis vaccination in infants and adolescents [3]. While coverage of childhood vaccinations is generally measured at 2 years of age, this metric does not capture delays in dose administration within the recommended programme. Our findings indicate that even in children and adolescents formally considered up to date with their immunisations, earlier administration could have prevented hospitalisation. A public health campaign aimed at both families and healthcare professionals, stressing not only the importance of vaccination but also the importance of timely administration, could help further reduce disease incidence and associated hospitalisations.

During the 2024 outbreak, the highest number of cases were seen among those aged 10–14 years. Among adolescents aged > 12 years, the vast majority had not yet received the second booster, despite eligibility from the age of 12 years onwards. These findings suggest two potential strategies to enhance immunisation coverage: firstly, to promote earlier administration of the fifth dose (adolescent booster), which is currently scheduled from 12 to 18 years of age; and secondly, to reduce the wide time frame (currently spanning 6 years) for this dose. One possible approach could be to recommend administration at 10–11 years of age.

Robust, year-round surveillance of all hospitalised cases is critical to identifying emerging signals such as those documented in the 2024 outbreak. Such data-driven approaches are essential to refine and adapt prevention strategies in a timely manner.

## Conclusions

To mitigate the resurgence of pertussis and prevent future outbreaks, a combination of measures should be adopted. These include strict adherence to the immunisation schedule, prioritising administration of vaccines at the earliest eligible opportunity, and consideration of advancing the adolescent booster dose. Strengthened surveillance systems are also essential to ensure timely adjustments to prevention strategies based on real-time epidemiological data.

## Data Availability

Data are available upon request.
